# Limitations of antimicrobial agent choice based on MIC value against Pseudomonas aeruginosa

**DOI:** 10.1099/jmm.0.002093

**Published:** 2025-11-14

**Authors:** Yurina Tamura, Masato Kawamura, Yuta Hoshino, Takumi Sato, Shigeru Fujimura

**Affiliations:** 1 Division of Clinical Infectious Disease and Chemotherapy, Tohoku Medical and Pharmaceutical University, Graduate School of Pharmacy, 4-4-1, Komatsushima, Aoba-ku, Sendai, 981-8558, Japan; 2 Department of Pharmacy, Tohoku Rosai Hospital, 4-3-21, Dainohara, Aoba-ku, Sendai, 981-8563, Japan

**Keywords:** antimicrobial resistance, broth microdilution method, MIC, mutant prevention concentration, mutant selection window, *Pseudomonas aeruginosa*

## Abstract

**Introduction.** In antibiotic chemotherapy, an antimicrobial agent is selected based on MIC, which is determined by a test method using the principle of the broth microdilution. However, it does not provide a viable count in the mutant selection window. In this study, we used turbidimetry and visual judgement to examine the validity of the selection of various antibiotics to treat *Pseudomonas aeruginosa* based on MIC determination after 24 h of incubation.

**Methods.** The antibiotics used in this study were piperacillin (PIPC), imipenem (IPM), meropenem, ciprofloxacin and amikacin (AMK). The strains used were 30 *P*. *aeruginosa* strains clinically isolated that were susceptible to all the antibiotics used, and the standard PAO1 strain. The viable count was measured after exposure for 3 and 24 h to therapeutic concentrations of various antibiotics, at which time turbidity was examined visually or by transmittance. In addition, the MPCs of IPM and PIPC were measured.

**Results.** In this study, 10²–10⁸ c.f.u. ml^−1^ of *P. aeruginosa* survived exposure to PIPC, IPM and AMK at concentrations 2.5–80 times the MIC despite high drug concentrations. No turbidity was observed in the culture medium. Furthermore, both IPM and PIPC showed high mutant prevention concentration (MPC), with 64.5% of strains in IPM and 10% of strains in PIPC showing intermediate or resistance after 24 h.

**Conclusions.** Choosing an appropriate antibiotic based on exceeding the MIC may be insufficient. While the PK/PD theory focuses on MIC, measuring MPC alongside MIC is urgent in clinical practice for optimal antibiotic selection.

## Introduction

The World Health Organization’s Global Action Plan on Antimicrobial Resistance (AMR) emphasizes optimizing antibiotic use. To combat AMR, appropriate antibiotic selection and dosing regimen design based on the pharmacokinetics (PK)/pharmacodynamics (PD) theory are essential [1]. PK/PD parameters include time above MIC, maximum plasma concentration (Cmax)/MIC and area under the blood concentration time curve (AUC)/MIC. It is especially important to know the exact MIC. In addition, the dosing regimen design should consider PK and mutant prevention concentration (MPC) to prevent the selection of antimicrobial-resistant mutants. The European Committee on Antimicrobial Susceptibility Testing and the Clinical and Laboratory Standards Institute consider the broth microdilution method as the standard method for MIC measurement [2, 3]. In this method, the MIC is determined by turbidimetry using an automated instrument (such as VITEK2^®^) or visual judgement. While both methods can determine an accurate MIC, neither provides a viable count in the mutant selection window (MSW).

Hagel *et al*. administered piperacillin (PIPC)/tazobactam to 48 patients with sepsis using therapeutic drug monitoring to achieve the optimal dose (target plasma concentration=4 MIC). They reported that at the end of treatment, the causative pathogen was eradicated in 56.3% of patients, while the remaining patients did not achieve a microbiological cure [4]. In short, even when bacteria are deemed susceptible, they may not be killed at therapeutic concentrations above the MIC. Thus, the MIC alone is an insufficient basis for appropriate antibiotic selection and dosing regimen design. In this study, we used turbidimetry and visual judgement to examine the validity of the selection of various antibiotics to treat *Pseudomonas aeruginosa* based on MIC determination after 24 h of incubation.

## Methods

### Bacterial strain

The strains used in this study were 30 of 236 *P*. *aeruginosa* strains clinically isolated from 16 general hospitals in Japan that were susceptible to PIPC (Sigma-Aldrich, MO), imipenem (IPM, FUJIFILM Wako Pure Chemical, Osaka), meropenem (MEPM, FUJIFILM Wako Pure Chemical), ciprofloxacin (CPFX, Sigma-Aldrich) and amikacin (AMK, FUJIFILM Wako Pure Chemical), and the standard PAO1 strain. MICs were determined by the broth microdilution method following CLSI [3]. The MIC ranges for the various antibiotics against the used strains were as follows: PIPC, 0.5–16 µg ml^−1^; IPM, 0.25–2 µg ml^−1^; MEPM, ≤0.06–1 µg ml^−1^; CPFX, ≤0.06–0.5 µg ml^−1^; and AMK, 0.25–2 µg ml^−1^.

### Bactericidal effect of the various antibiotics

The viable count was measured after exposure to therapeutic concentrations of various antibiotics. That is, the bacterial strain was adjusted to ~5×10^5^ c.f.u. ml^−1^ in Mueller–Hinton broth (MHB), and each antibiotic was added to 1 ml of this culture medium to achieve the Cmax (PIPC: 80 µg ml^−1^; IPM: 20 µg ml^−1^; MEPM: 20 µg ml^−1^; CPFX: 2.5 µg ml^−1^; and AMK: 5 µg ml^−1^) when administered intravenously at the following doses: PIPC at 2 g, IPM at 0.25 g, MEPM at 0.25 g, CPFX at 0.2 g and AMK at 0.1 g. After incubation at 35 °C, the viable count was measured 3 and 24 h later. Then, MICs of the viable bacteria were measured.

### Visual transmittance

The viable count that all five microbiologists determined visually that the sample was turbid and the transmittance (T) at that time w investigated. T was calculated from absorbance (A) using the iMark Microplate Reader (Bio-Rad).


$$A=-log\frac{T}{100}$$

The relative transmittance (T′), when the transmittance of only MHB (T_MHB_) was 100%, was obtained from the following equation:


$$T`=\frac{T}{T_{MHB}}\times 100$$

### Mutant prevention concentration

The MPCs of IPM and PIPC in the wild-type strains that were found to survive after 24 h of exposure to antibiotics were measured by the agar plate dilution method. Specifically, 100 µl of bacterial suspension adjusted to >1×10^11^ c.f.u. ml^−1^ was spread on an antibiotic-containing agar plate, and the minimum concentration at which no colony formation was observed after incubation at 35 °C for 48 h was defined as the MPC [5].

## Results

### Bactericidal effect of the various antibiotics

**Fig. 1.**
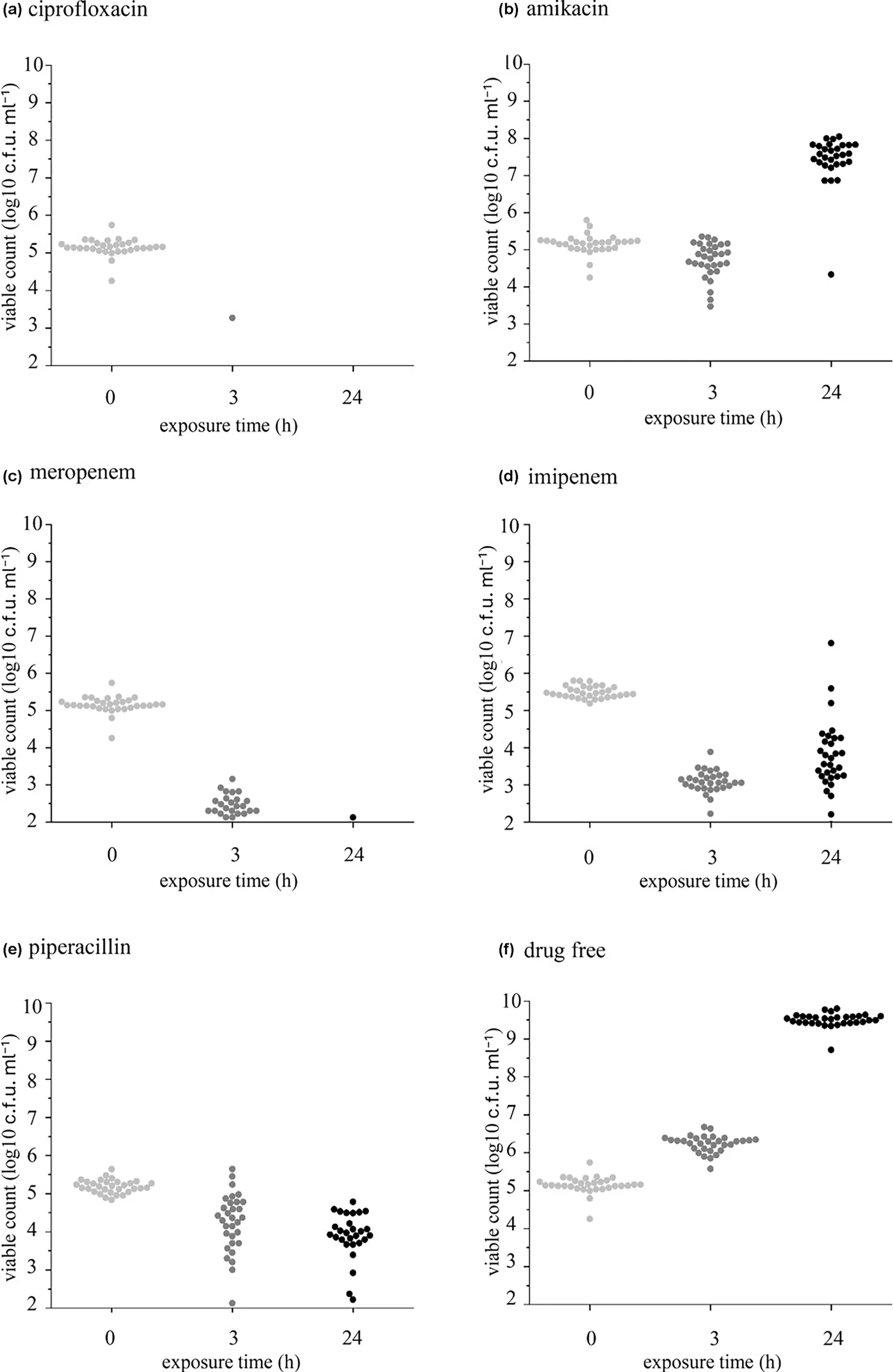
Changes in viable count after exposure to various antibiotics. The viable count was measured after exposure to A: CPFX, B: AMK, C: MEPM, D: IPM and E: PIPC for 3 and 24 h, respectively. Panel F shows the viable count without antibiotics.

CPFX killed 96.8% of the strains after 3 h, and all the strains were killed at 24 h (Fig. 1a). In contrast, after 24 h of AMK exposure, 28 of 31 strains (90.3%) remained viable counts of 10^6^–10^8^ c.f.u. ml^−1^ (Fig. 1b). The 28 wild-type strains had AMK MICs of 1–2 µg ml^−1^, whereas 24 h after exposure to AMK, the MICs of 16 strains (57.1%) increased by one to two tubes. Among the β-lactams, 30 of the 31 strains (96.8%) were below the detection limit within 24 h of MEPM exposure (Fig. 1c). Meanwhile, IPM reduced the viable count in all strains after 3 h, although only one strain was completely killed after 24 h, with 30 strains (96.8%) showing viable counts of 10^2^–10^6^ c.f.u. ml^−1^ (Fig. 1d). Of these 30 strains, 29 showed increased MICs, of which 14 (48.3 %) were intermediate (MIC=4 µg ml^−1^) and six (20.7%) were resistant (MIC=8 µg ml^−1^). Similarly, with PIPC, 28 strains (90.3%) survived at 10^2^–10^5^ c.f.u. ml^−1^ after 24 h (Fig. 1e), among which two strains were intermediate (MIC=32 µg ml^−1^) and one was resistant (MIC=128 µg ml^−1^).

### Visual transmittance

**Fig. 2.**
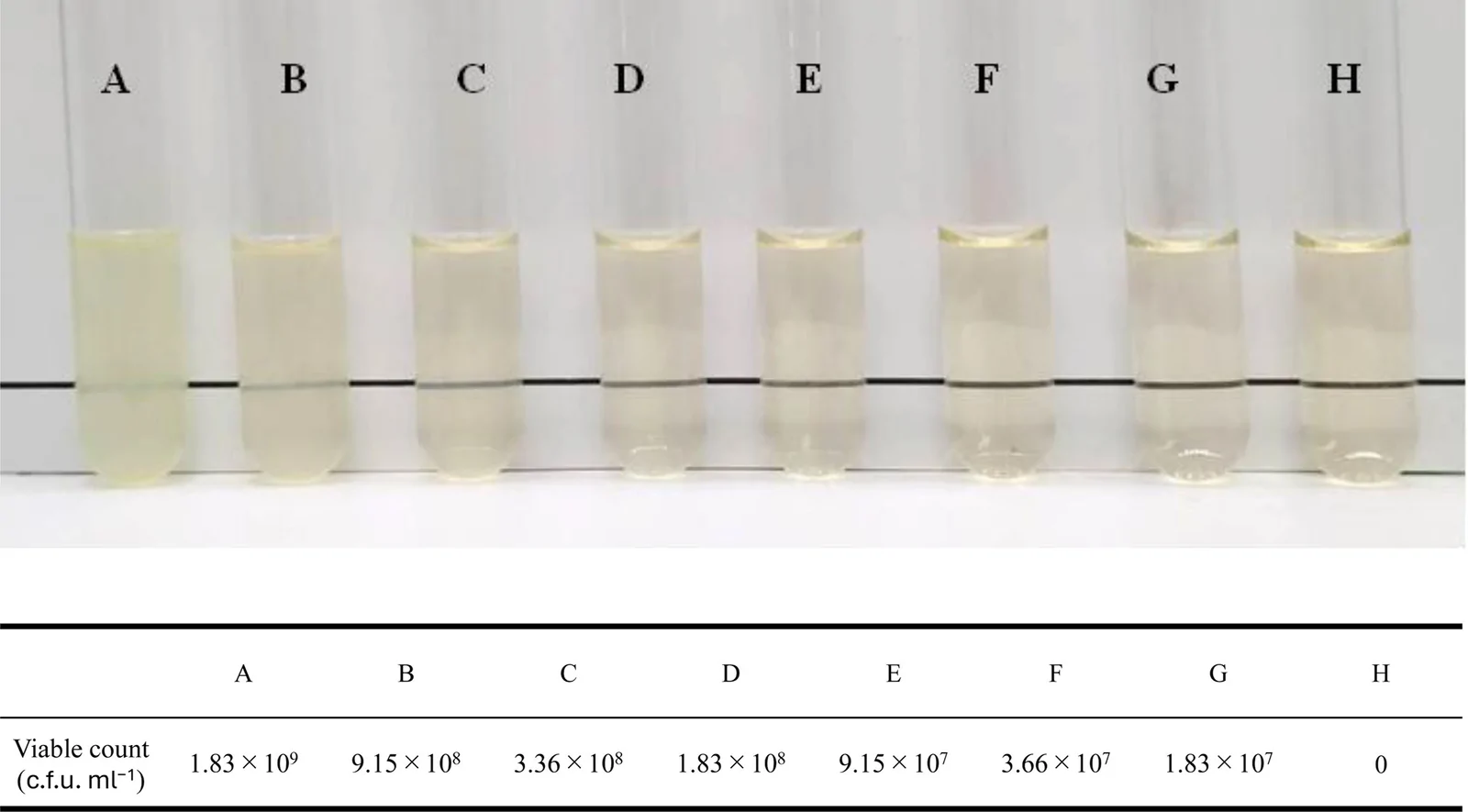
Visual judgement of turbidity in test tubes containing MHB and viable count. The black line is placed in the background of the test tubes, and it becomes less visible when the MHB in the test tube becomes turbid. In this examination, all the microbiologists judged test tubes A and B as turbid. In contrast, all the microbiologists judged test tubes F, G and H as non-turbid (clear).

The T′ of the culture medium adjusted to ~5×10^5^ c.f.u. ml^−1^ with antibiotic-free MHB was 99.4% and was clear visually. After 24 h of incubation without the antibiotics, the cultures showed an average viable count of 3.38×10^9^ c.f.u. ml^−1^ (Fig. 1f), and all the microbiologists visually judged the cultures to be turbid (T′: 38.1%) (Fig. 2). The minimum viable count determined to be turbid in this study was 3.95×10^8^ c.f.u. ml^−1^ (T′: 74.9%). Meanwhile, the maximum viable count determined to be free of turbidity was 4.44×10^7^ c.f.u. ml^−1^ (T′: 98.7%). When the viable count was below the detection limit (1.00×10² c.f.u. ml^−1^) after exposure to the antibiotics for 24 h, T′ was 98.3–100% (average 99.5%). The average of T′ after 24 h of exposure to each antibiotic was as follows: PIPC: 99.5%; IPM: 99.5%; MEPM: 99.8; CPFX: 99.5%; and AMK: 97.0%, respectively, and all were visually judged to be clear with no visual turbidity.

### Mutant prevention concentration

The MPC range of IPM for 30 strains was 32–256 µg ml^−1^, whereas that of PIPC for all 28 strains was >256 µg ml^−1^.

## Discussion

In this study, we investigated the bactericidal effects of various antibiotics against *P. aeruginosa* at therapeutic concentrations based on the MIC. For the concentration-dependent antibiotics, CPFX completely killed *P. aeruginosa* after 24 h exposure at concentrations 5–42 times the MIC, whereas AMK at concentrations 2.5–5 times the MIC did not show a bactericidal effect. The reason behind AMK was thought to be the concentration not being sufficient, because it was reported that Cmax/MIC≥8 is required to kill *P. aeruginosa* [6, 7]. Notably, AMK-susceptible *P. aeruginosa* showed an increased viable count to an average of 3.77×10^7^ c.f.u. ml^−1^, despite being visually clear in the culture at concentrations above the MIC. While it should be noted that T′ cannot reliably detect increased viable count, it allows for lower-level detection as compared to visual judgement. And then, judging the bactericidal effect solely based on MIC from the broth microdilution method may overestimate the bactericidal effect of AMK, suggesting caution in its use.

The bactericidal effect of carbapenems was greater with MEPM than with IPM, which was thought to be due to differences in the bacterial uptake. IPM is taken up into the bacterial cell via OprD and OpdP, whereas MEPM is transported not only through OprD and OpdP but also via additional porins, such as OprF and OprE [8, 9]. Furthermore, in both Europe and Japan, the resistance rate of *P. aeruginosa* to IPM was reported to be higher than that to MEPM [10–12], which may be attributed to this intrinsic mechanism.

Furthermore, despite being exposed to concentrations 5–80 times the MIC, both IPM and PIPC showed insufficient bactericidal effects, with 64.5% of strains in IPM and 10% of strains in PIPC showing intermediate or resistance after 24 h. Due to high MPC, the concentrations of these antibiotics remained within the MSW, suggesting the selection of antimicrobial-resistant mutants. The increase in MICs within the MSW, which cannot be detected by visual judgement or T′, poses a significant concern. Therefore, we suggest that MPC, as well as MIC, should be focused.

In summary, our findings indicate that relying on MIC alone by the broth microdilution method may overestimate the effectiveness of antibiotics and underestimate the potential for resistance development. We believe that the inclusion of MPC measurement alongside MIC may support more effective treatment strategies.

In summary, *P. aeruginosa* survived even after 24 h of exposure to AMK, PIPC and IPM at concentrations exceeding their MICs, and an increase in MIC was observed in some strains. Cunha *et al*. reported that gentamicin, tobramycin, ceftazidime and IPM have high AMR potential due to their susceptibility to resistance development in *P. aeruginosa* [13]. The results of this study support their findings. In other words, *P. aeruginosa* can survive within the MSW of aminoglycosides and some of β-lactam antibiotics, indicating that careful attention should be paid to the emergence of resistant strains when using these antibiotics.

## Abbreviations

AMK: amikacin
AMR: antimicrobial resistance
AUC: area under the blood concentration time curve
Cmax: maximum plasma concentration
CPFX: ciprofloxacin
IPM: imipenem
MEPM: meropenem
MHB: Mueller–Hinton broth
MPC: mutant prevention concentration
PD: pharmacodynamics
PD: pharmacodynamics
PIPC: piperacillin
PK: pharmacokinetics
